# Proteotoxic Stress as an Exploitable Vulnerability in Cells with Hyperactive AKT

**DOI:** 10.3390/ijms222111376

**Published:** 2021-10-21

**Authors:** Mahamat Babagana, Lorin R. Brown, Hannah Z. Slabodkin, Julia V. Kichina, Eugene S. Kandel

**Affiliations:** Roswell Park Comprehensive Cancer Center, Department of Cell Stress Biology, Buffalo, NY 14263, USA; mahamat.i.babagana.mil@mail.mil (M.B.); lorinbro@buffalo.edu (L.R.B.); hzs@stanford.edu (H.Z.S.); Julia.Kichina@roswellpark.org (J.V.K.)

**Keywords:** heat shock, AKT, PTEN, XBP1, HSF1, HSP70, HSP90

## Abstract

Hyperactivity of serine-threonine kinase AKT is one of the most common molecular abnormalities in cancer, where it contributes to poor outcomes by facilitating the growth and survival of malignant cells. Despite its well-documented anti-apoptotic effects, hyperactivity of AKT is also known to be stressful to a cell. In an attempt to better elucidate this phenomenon, we observed the signs of proteotoxic stress in cells that harbor hyperactive AKT or have lost its principal negative regulator, PTEN. The activity of HSF1 was predictably elevated under these circumstances. However, such cells proved more sensitive to various regimens of heat shock, including the conditions that were well-tolerated by syngeneic cells without AKT hyperactivity. The sensitizing effect of hyperactive AKT was also seen in HSF1-deficient cells, suggesting that the phenomenon does not require the regulation of HSF1 by this kinase. Notably, the elevated activity of AKT was accompanied by increased levels of XBP1, a key component of cell defense against proteotoxic stress. Interestingly, the cells harboring hyperactive AKT were also more dependent on XBP1 for their growth. Our observations suggest that proteotoxic stress conferred by hyperactive AKT represents a targetable vulnerability, which can be exploited by either elevating the stress above the level tolerated by such cells or by eliminating the factors that enable such tolerance.

## 1. Introduction

Serine-threonine kinase AKT is a key component of multiple signaling pathways in mammalian cells [1]. In human cells, AKT is represented by three major isoforms (AKT1, AKT2, AKT3), which differ in their exact patterns of expression and biological roles, but share a very high degree of homology and a similar mode of activation. In the classical scheme, its interaction with phospholipid products of phosphatidylinositol 3-kinase (PI3K) attracts AKT to the plasma membrane, where it undergoes phosphorylation and full activation [1,2]. Active AKT can phosphorylate a multitude of proteins, affecting transcription, translation, metabolism, survival, motility, proliferation, etc. [1]. While the enzymes from the PI3K family contribute to the activation of AKT via the production of phospholipids, the reverse reaction catalyzed by the Phosphatase and TEnsin Homolog (PTEN) phosphatase serves to curtail AKT activity [3]. Importantly, hyperactivation of AKT is one of the most common molecular pathologies in cancer [2,4]. It can result from the pathological activation of PI3K, either via direct mutation or amplification of the genes for PI3K catalytic subunits or via the pathological activation of its upstream regulators, such as receptor tyrosine kinases, G-protein coupled receptors, or RAS family members. It can also result from the loss of PTEN function, and this abnormality is highly prevalent in human malignancies [5]. Hyperactive AKT contributes to the survival and therapy resistance of cancer cells [1,6]. AKT attenuates the efficacy of targeted therapies [7] and can alter the cell response to DNA damage and facilitate the acquisition of mutations [8]. It also contributes to epithelial–mesenchymal transition and metastasis [9] and cooperates with other pathways in enabling oncogenic transformation [10]. AKT inhibits tumor-suppressive transcription factors from the FOXO family and activates several transcription factors with well-known pro-tumorigenic properties [1]. The heightened demands of rapidly proliferating cancer cells are supported by the AKT-dependent enhancement of translation and metabolism [11,12]. Unsurprisingly, pharmacological targeting of AKT has long been sought as an anti-cancer intervention. Unfortunately, this approach has met with very limited success. According to multiple earlier studies, AKT inhibitors have shown little to no efficacy [13,14,15,16,17,18] despite pronounced toxicity [19]. The latter is not surprising, considering the widespread role of AKT in normal organismal functions [1]. There are some encouraging recent results from trials of newer inhibitors and their combinations with other agents [20], and, despite the earlier setbacks, an AKT-targeting pharmacological intervention may still become a reality in the future.

Interestingly, akin to many other oncogenes, hyperactivation of AKT is stressful to a normal mammalian cell. Hypothetically, this phenomenon may provide an exploitable vulnerability in pre-cancerous or cancerous cells with elevated AKT activity. Conceivably, augmenting the type of damage already imparted by AKT would be especially detrimental to such cells, overwhelming the already strained protective mechanisms. Furthermore, such cells, compared to their normal counterparts, may be more dependent for survival on the stress-mitigating pathways, so that transient inhibition of the latter might be employed for cancer therapy or prevention. This promise of the targeted elimination of cells with the specific molecular abnormality underscores the need for an improved understanding of the types and the underlying mechanisms of stress induced by hyperactive AKT.

The accumulation of damaged or misfolded proteins endangers cell viability [21]. Under normal conditions, upwards of 30% of all newly synthesized proteins are targeted for immediate recycling, presumably due to errors in translation and folding [22]. This burden can be further elevated by various intrinsic and extrinsic impacts, resulting in proteotoxic stress in an affected cell, which then becomes dependent on the mobilization of specialized stress response mechanisms for survival. For example, hyperthermia, among other consequences, leads to the unfolding of existing proteins [23]. The classic heat shock response is orchestrated by transcription factor HSF1, which elevates the production of chaperones that facilitate the refolding or recycling of affected molecules [23]. Furthermore, the accumulation of misfolded proteins in the endoplasmic reticulum (“ER stress”) triggers a specialized and complex unfolded protein response through PERK, IRE1α, and ATF6 [24,25]. Activation of these sensors collectively attenuates the general biosynthesis of new proteins while facilitating ER-associated protein degradation and the production of factors that promote polypeptide refolding. In particular, ATF6 facilitates the enhanced transcription of the XBP1 gene [26], while the multifunctional enzyme IRE1α catalyzes the unconventional splicing of the XBP1 mRNA [27], creating a suitable open reading frame for the synthesis of XBP1s. The shortened splicing product (sometimes called “XBP1s”) codes for a functional transcription factor, which coordinates the expression of the molecules that facilitate the refolding or recycling of damaged proteins, and is a major determinant of stress resistance in cells subjected to moderate proteotoxic stress [28]. Importantly, a cell has a limited capacity to deal with proteotoxic stress. When this capacity is overwhelmed, the pro-survival cellular program converts into a program of cell death [24,29].

Proteotoxic stress is a common feature of cancer cells. Therapeutic strategies to increase its lethality by either facilitating additional protein damage or inhibiting detoxifying mechanisms have garnered some evidence of success in clinical and pre-clinical studies of cancer therapy and prevention [25]. In this current report, we explore the relationship between hyperactivation of AKT and the heat shock response in non-tumor-derived cells, which are less likely to carry secondary adaptive alterations acquired in the course of a prolonged tumor evolution. 

## 2. Results

### 2.1. Signs of Activated Heat Shock Response Accompany Hyperactivation of AKT

**Figure 1:**
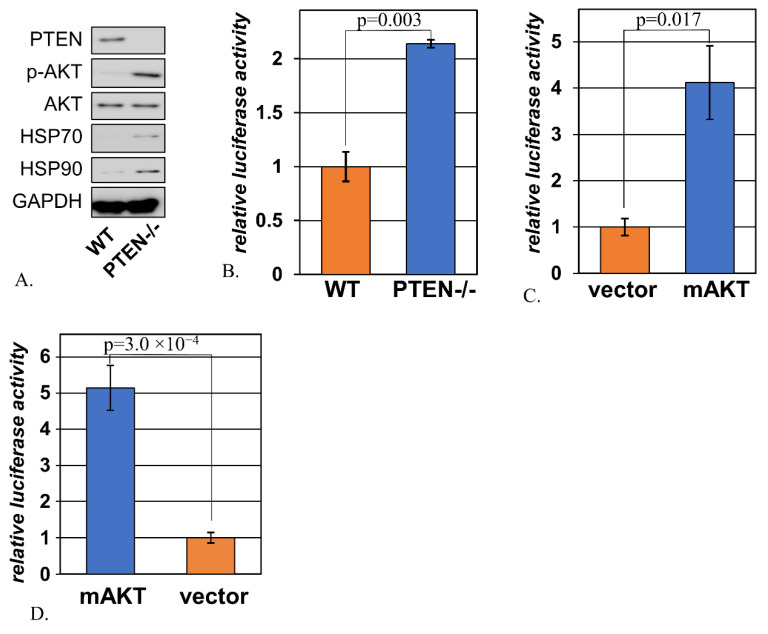
**The loss of PTEN and activation of AKT trigger the heat shock response.** (**A**). The lysates from MEF PTEN-/- and MEF WT cells were probed by immunoblotting with the indicated antibody. (**B**). The activity of the HSF1-driven luciferase reporter was measured upon transient transfection into MEF WT and MEF PTEN-/- cells. The data from triplicate experiments are shown relative to the average values in MEF WT. (**C**). The activity of the HSF1-driven luciferase reporter was measured upon transient co-transfection into MEF-AG cells with either an activated form of mouse AKT1 (mAKT) or the corresponding vector control. The data from triplicate experiments is shown relative to the average values in vector-transfected cells. (**D**). The activity of the HSF1-driven luciferase reporter was measured upon transient co-transfection into human 293T cells with either an activated form of mouse AKT1 (mAKT) or the corresponding vector control. The data from triplicate experiments are shown relative to the average values in vector-transfected cells.

The loss of PTEN is a common mode of activation of AKT in cancer [5]. This event often marks the progression from a pre-malignant to a malignant lesion, or the evolution of a cancer towards a more therapy-resistant and metastasis-prone phenotype [30]. In order to examine cell stress induced by PTEN loss without the confounding contributions of additional alterations selected for during tumor evolution, we relied on immortalized mouse embryonic fibroblasts that carried a “floxed” version of the Pten gene. In these cells, Cre recombinase can quickly and efficiently excise from the Pten gene its fifth exon, which normally encodes the catalytic domain of the PTEN protein [31]. Indeed, the derivatives of these cells expressing Cre (designated “MEF PTEN-/-”) had markedly reduced levels of PTEN and much higher levels of active (phosphorylated) AKT in comparison to the cells transduced with the corresponding empty vector (designated “MEF WT”) (Figure 1A). Notably, this was accompanied by elevated expression of Hsp70 and Hsp90 (Figure 1A). Both of these chaperones are classical markers of the heat shock response, and the corresponding genes are transcriptional targets of HSF1 [32,33]. Accordingly, the transcriptional activity of HSF1 was increased by the removal of PTEN (Figure 1B). This effect was recapitulated in MEF-AG cells by transfection of a constitutively active AKT mutant (Figure 1C). This effect was not exclusive to mouse embryonic fibroblasts: activated AKT similarly increased HSF1 activity in human kidney epithelial cells HEK293 (Figure 1D).

### 2.2. Cells with Activated AKT Are Hypersensitive to Hyperthermia

We considered two possible causes for elevated HSF1 activity in the presence of hyperactive AKT. First, AKT might activate HSF1 through a signal transduction mechanism, which merely mimics the signaling during proteotoxic stress, without any actual damage to the cell. In this case, one would expect increased resistance of such cells to a proteotoxic or heat shock challenge because the chaperones capable of mitigating such an impact are already present in the cell at elevated levels. Second, increased signaling by AKT may cause actual damage to a cell, which would thence trigger the observed HSF1 activation. Importantly, a cell has a limited capacity to counteract proteotoxic stress, and the continuous increase in the severity of damage converts the protective response into a cell death program [29]. Thus, in the second scenario, one would expect that an additional proteotoxic impact might be more damaging in combination with hyperactive AKT, because the latter places extra strain on the stress resistance mechanisms and moves the cell closer to the threshold of viability. Therefore, we sought to investigate the effect of hyperactive AKT on resistance to supplementary stress in our experimental model.

**Figure 2:**
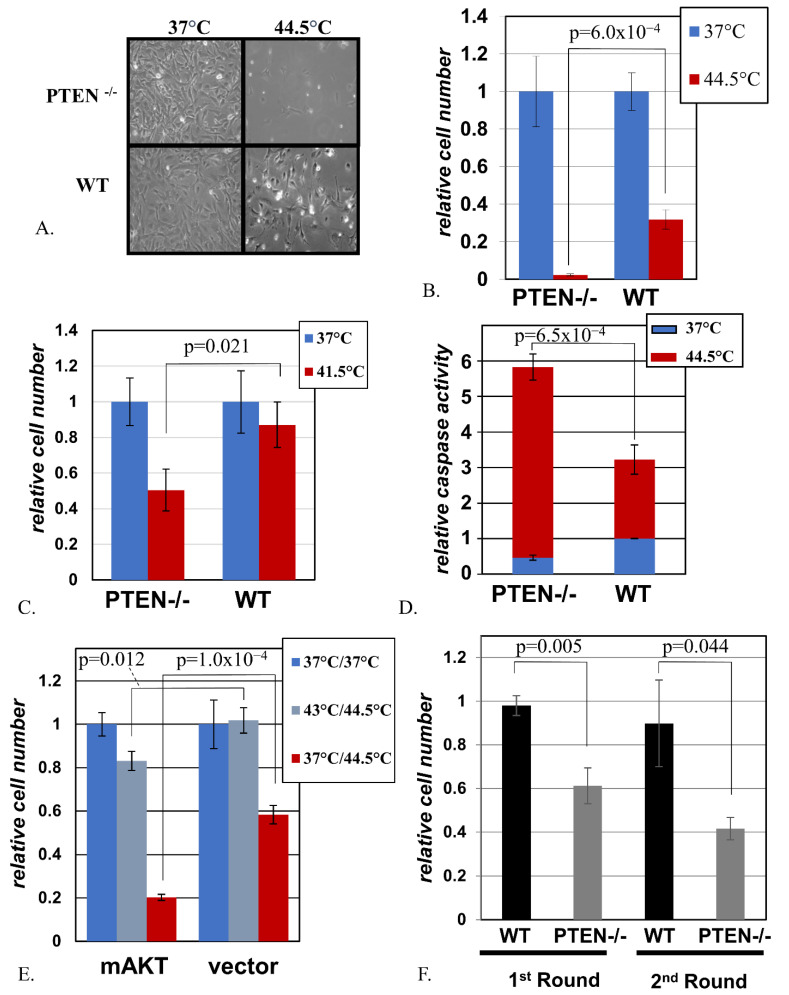
**The loss of PTEN and activation of AKT enhance sensitivity to hyperthermia.** (**A**). MEF PTEN-/- and MEF WT were exposed to the indicated temperatures for 30 min, allowed to recover in normal growth conditions for 24 h, and photographed. (**B**). The number or remaining MEF PTEN-/- and MEF WT cells treated as in A was compared 48 h after the heat shock. The data from triplicate experiments for each cell line are shown normalized to the average values in the respective control (37 °C) populations. (**C**). MEF PTEN-/- and MEF WT were exposed to the indicated temperatures for 2 h and then analyzed as in B after recovering for 48 h. The data from triplicate experiments for each cell line are shown normalized to the average values in the respective control (37 °C) populations. (**D**). The activity of caspase 3 was measured in MEF PTEN-/- and MEF WT cell populations treated as in A. The data from triplicate experiments for each cell line are shown normalized to the average values in the control (37 °C) populations of MEF WT. (**E**). MEF-AG harboring either mAKT or the respective empty vector (EV) were subjected to a pre-treatment at either 43 °C or 37 °C for 30 min, followed 5 h later by a 30 min heat shock at 44.5 °C. Then, 48 h later, the numbers of remaining cells were scored. For each cell line, the values from triplicate experiments are plotted relative to those of the respective control populations grown without hyperthermia (“37 °C/37 °C”). (**F**). Triplicate cultures of MEF PTEN-/- and MEF WT were heat-shocked at 41.5 °C for 2 h and, 48 h later, the remaining cells were scored (“1st round”). A parallel set of three heat-shocked cultures of each cell line was allowed to recover under normal culture conditions. A week later, the recovered cells were subjected to a repeat of the heat shock treatment, and the remaining cells were scored 48 h later (“2nd round”). For each cell variant, the data on the remaining cells from triplicate cultures after each round of treatment are shown after normalization to the average values from their respective control populations (also triplicates) grown at 37 °C.

In accordance with the prediction from the second scenario, we observed that the loss of PTEN was associated with increased sensitivity to heat shock under various conditions of treatment (Figure 2A–C,F). In accordance with the well-known antiapoptotic role of the AKT pathway, PTEN-deficient cells displayed a reduced level of apoptosis during routine culture; however, the same cells greatly exceeded their MEF WT counterparts in the induction of apoptosis under hyperthermia (Figure 2D). 

AKT proteins are important, but not exclusive, downstream effectors of PTEN [34]. We thus investigated whether activation of AKT alone is sufficient to mimic the sensitizing effect of PTEN inactivation. Indeed, the introduction of a constitutively active mutant of AKT into spontaneously immortalized mouse embryonic fibroblasts MEF-AG strongly sensitized the cells to heat shock (Figure 2E). This sensitizing effect of constitutive AKT was reproduced in yet another MEF cell line, p53-deficient MEF-P53-/- (Suppl. Figure S1). 

Of note, pre-treatment of cells with sub-lethal hyperthermia is known to protect cells from a closely followed treatment with otherwise lethal heat shock [35]. This mechanism is functional in MEF WT cells (Figure 2E). However, despite the protection rendered by this pre-treatment, cells harboring activated AKT remained more sensitive than their vector-transduced counterparts (Figure 2E). 

Interestingly, under our experimental conditions, MEF PTEN-/- cells that survived and recovered after one round of heat shock remained more heat-sensitive than MEF WT cells that underwent the same treatment (Figure 2F). This suggests a possibility for a more complete elimination of cells with hyperactive AKT upon repetitive application of hyperthermia, which has no more than minimal toxicity to the cells with normal AKT status. 

### 2.3. Activated AKT Sensitizes Cells to Hyperthermia in the Absence of HSF1

**Figure 3:**
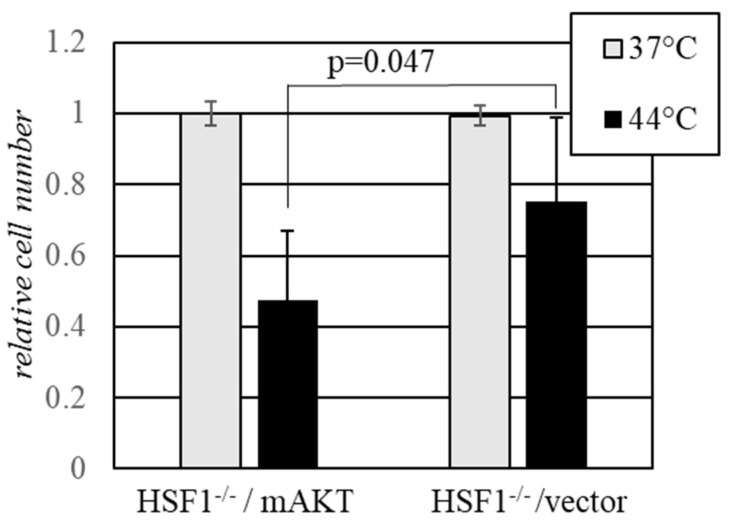
Activated AKT sensitizes to heat shock independently of HSF1. HSF1-deficient MEF (HSF-/-) harboring either mAKT or the corresponding empty vector were treated for 30 min at the indicated temperatures. The remaining cells were scored after 48 hours of recovery under normal growth conditions. The data from triplicate experiments for each cell line are shown normalized to the average values in the respective control (37 °C) populations.

Transcription factor HSF1 is believed to be the central coordinator of the heat shock response in mammalian cells [23]. It has been reported that HSF1 may be a subject of regulation and direct phosphorylation by AKT independently of heat shock [36]. We sought to investigate whether the sensitizing effect of AKT towards heat shock, as observed in our experiments, requires HSF1. We thus tested the effect of AKT hyperactivity on the sensitivity to heat shock in HSF1-deficient mouse embryonic fibroblasts. Akin to other MEFs tested in our study, the HSF1-deficient cells were sensitized to heat shock by the expression of hyperactive AKT (Figure 3), confirming that the observed sensitization phenomenon is HSF1-independent. 

### 2.4. Activated AKT Increases Cell Dependence on XBP1

**Figure 4:**
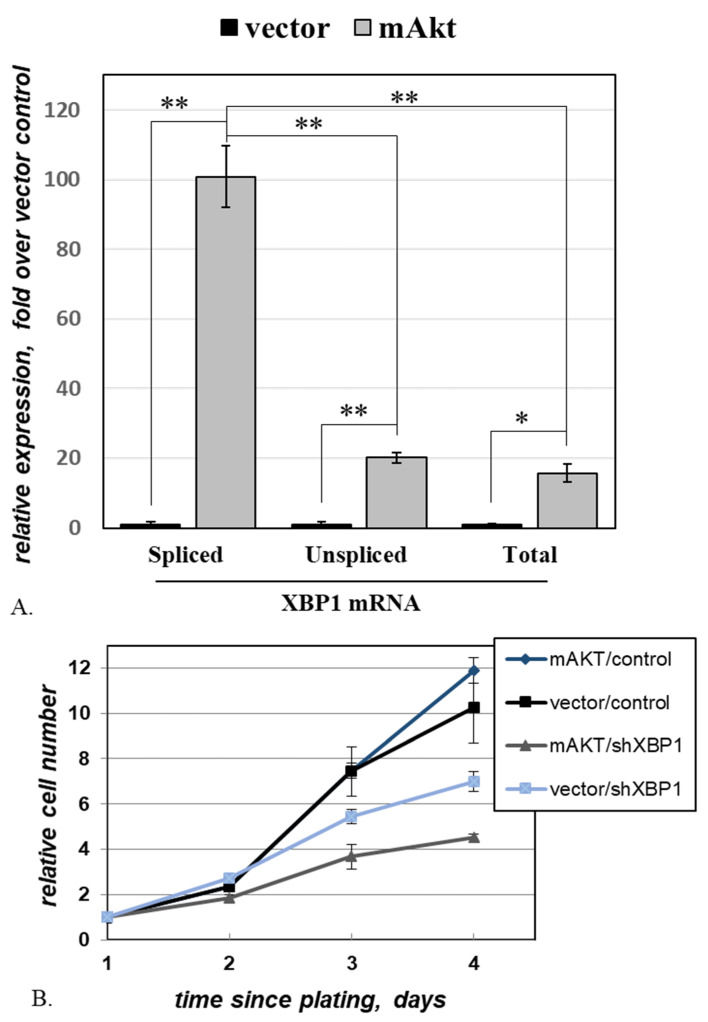
**The heightened dependence on XBP1 in the presence of activated AKT.** (**A**). MEF-AG cells transduced with mAKT or the respective empty vector were assayed for the expression of the indicated XBP1 transcripts using quantitative RT-PCR. After normalization to the GAPDH signal in the same samples, the values for XBP1 variants in each cell line were plotted relative to the average value in the empty vector control, and results analyzed using an ANOVA Tukey’s multiple comparisons test. Error bars represent standard deviations of triplicates. “*”-*p* = 5.0 × 10^−3^. “**”-*p* < 1.0 × 10^−4^. (**B**). MEF-AG cells harboring either mAKT or the respective empty vector were transduced with either the XBP1 shRNA or the respective control construct and plated at the same density for growth rate comparison. The remaining cells for each cell line were scored at the indicated time points after plating, and the values were plotted relative to those on day 1. Quadruplicate cultures of each cell variant were analyzed for each time point.

In a cell subjected to proteotoxic stress, an active form of transcription factor XBP1 (XBP1s) accumulates due to the concomitant activation of ATF6, which increases the transcription of the XBP1 gene [26], and IRE1α, which splices the XBP1 mRNA [26,27]. We used quantitative RT-PCR to assay the changes in the levels of XBP1 transcripts upon the introduction of activated AKT (Figure 4A). The observed pattern is consistent with what is predicted for cells under proteotoxic stress: in the presence of mAKT, all assayed transcript variants increased, with a disproportionally high increase recorded for the spliced variant (Figure 4A). 

As XBP1s plays a protective role under modest proteotoxic stress, we explored whether mAKT-harboring cells depend on this protection for their continuous survival. We followed the growth of MEF-AG cells, harboring either an mAKT-expressing construct or the respective vector control, and additionally transduced with either an shRNA targeting XBP1 or a control non-targeting shRNA (Figure 4B). In this experiment, the growth of vector-transduced cells was not significantly affected by the presence of mAKT. In contrast, while the knockdown of XBP1 affected both variants of MEF-AG, it was significantly more detrimental against the background of mAKT (*p* = 8.0 × 10^−4^ for the interaction between mAKT and XBP1 shRNA). The heightened dependence on XBP1 imparted by hyperactive AKT points to a potentially exploitable vulnerability in cancerous and pre-cancerous cells where AKT is activated.

### 2.5. Activated AKT Sensitizes a Human Melanoma Cell Line to Hyperthermia

**Figure 5:**
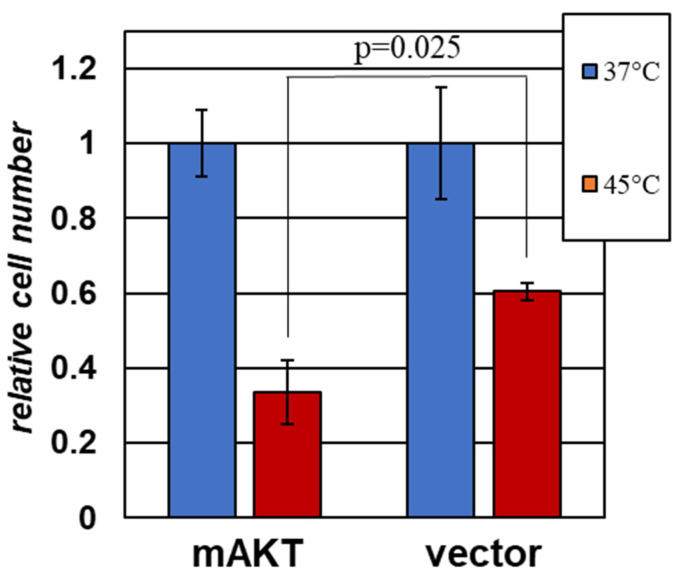
**A human melanoma cell line is sensitized to hyperthermia upon expression of hyperactive AKT.** A375 melanoma cells harboring either mAKT or the corresponding empty vector were treated for 60 min at the indicated temperatures. The remaining cells were scored after 24 h of recovery under normal growth conditions. The data from triplicate experiments for each cell line are shown normalized to the average values in the respective control (37 °C) populations.

In order to verify whether hyperactive AKT can sensitize human cancer-derived cells to hyperthermia, we relied on A375 human melanoma cells that were engineered with either a constitutive active variant of AKT (mAKT) or the corresponding empty vector control (pBabePuro). Ectopic expression of this AKT mutant has been shown to increase the resistance of A375 cells to targeted therapies [37]. In contrast, the mAKT-expressing cells were markedly more sensitive to hyperthermia (Figure 5). We concluded that the sensitizing effect of activated AKT is not limited to mouse fibroblasts and may be observed against the background of human cancer-derived cells. 

## 3. Discussion

Our observations presented herein provide evidence of proteotoxic stress imparted by elevated activity of AKT (Figure 1 and Figure 4A). Furthermore, they demonstrate that this stress is associated with the heightened dependence of cells on the function of XBP1, whose known role is to mitigate proteotoxicity. Accordingly, hyperthermia, which is a classical trigger of proteotoxic heat shock, was especially detrimental to the cells that carried a hyperactive AKT variant or lacked PTEN, the key negative regulator of AKT activity. 

Our results show that the lack of PTEN and the ensuing activation of AKT are associated with increased apoptosis in heat-shocked cells. It is possible, however, that the net reduction in cell numbers in treated cultures also includes the cumulative effects on cell proliferation, autophagy, and non-apoptotic cell death. 

A prior report has suggested that AKT may have a direct effect on HSF1 activity: under normal growth conditions, in the absence of heat shock, AKT increased HSF1 activity, which, in turn, increased the expression of certain markers of epithelial–mesenchymal transition (EMT) in breast carcinomas [36]. It is well-known that HSF1 in cancer cells exerts functions unrelated to the heat shock response [38], so the regulation of HSF1 in the context of EMT may or may not have a direct effect on thermotolerance.

It was also reported that high AKT activity contributes to the upregulation of heat shock proteins in the contexts of multiple myeloma [39] and chronic lymphocytic leukemia [40]. While the effect was originally attributed to the direct regulation of HSF1 by the AKT pathway, the proteotoxic consequences of AKT expression were not explored in these studies. Interestingly, in both scenarios, the overexpression of the heat shock proteins was critically required for the survival of the cells under regular growth conditions [39,40], which is consistent with the existence of proteotoxic stress in these cells. 

The role of AKT as a sensitizing factor may appear counterintuitive. Indeed, the activation of this enzyme is a well-known anti-apoptotic event, which mediates the pro-survival functions of growth factors [41,42] and increases the resistance of cells to a multitude of potentially cytotoxic impacts [8,43]. Nevertheless, our observation is not without precedent. There are ample examples of oncogenic factors, which often contribute to tumor growth and survival, decreasing the survival and proliferative potential of cells upon hyperactivation or under specific stress conditions [44,45,46,47]. In particular, it has been long known that hyperactivity of AKT is stressful, especially to normal cells [48,49,50]. This stress has been linked to an increase in the production of reactive oxygen species (ROS), which may be attributed to the inactivation of FOXO transcription factors and the resulting downregulation of detoxifying enzymes [48,49], coupled with increased oxygen consumption [49], which ensues from enhanced oxidative phosphorylation [42]. Alternatively, it was proposed that AKT may cause cell stress through the activation of mTOR [50]. Importantly, it was argued that this phenomenon does not require DNA damage [50], which is consistent with our observation of proteotoxic stress upon AKT hyperactivation. One may envision several avenues of how proteotoxic stress arises upon activation of AKT. The oxidative damage to proteins due to heightened ROS levels and the accumulation of misfolded proteins due to an increase in the translation rate are trivial suggestions. However, more complicated mechanisms, such as altered proteasomal activity or altered protein traffic through Golgi, cannot be ruled out at this time. It is also possible that multiple AKT-dependent processes contribute to the ensuing proteotoxic stress at the same time.

The stress-inducing property of AKT coupled to the high frequency of activation of this oncogene in human malignancies and pre-cancerous lesions provides an attractive avenue for the selective targeting of cancer cells. One approach to this problem is by augmenting the type of damage that is already induced by AKT, so as to bring it over the lethal threshold. In an earlier proof-of-principle study, it was shown that the agents that increase ROS production are preferentially toxic to the cells with activated AKT [49]. Unfortunately, supraphysiological doses of the agents that directly increase intracellular ROS (e.g., hydrogen peroxide) are toxic, mutagenic, and carcinogenic [51], while the agents that increase ROS indirectly (phenethyl isothiocyanate, rapamycin, etc.) have a multitude of other effects, which makes it challenging to attribute their therapeutic action to the selective targeting of an AKT-related vulnerability. In this regard, heat shock using the temperatures that are well-tolerated by normal cells, as was done in some of our experiments, suggests an attractive alternative strategy to target cells with hyperactive AKT in vivo. While this did not cause the complete elimination of the targeted population, the latter remained hypersensitive to repeated treatment. Thus, it might be possible to achieve more comprehensive control of the target population by the repetitive application of relatively modest hyperthermia. It is also worth mentioning that in an immunocompetent host, the selective thermal destruction of a tumor is likely to potentiate an immune response, which would further augment tumor control [52]. Of note, our observations suggest that the presence of p53 is not essential for AKT-mediated sensitization, as the latter occurs in p53-deficient cells (MEF-P53-/-) and in cells harboring a dominant negative form of p53 (MEF PTEN-/-). This observation is important for the prospects of exploiting this vulnerability in pre-malignant or malignant lesions, since p53 is commonly lost or inactivated in human cancers. An additional feature of thermal therapy is that it could be applied either to large areas or in a precisely targeted manner.

Another approach toward exploiting AKT-induced stress is by targeting the protective mechanisms that otherwise enable the tolerance of such a stress in an affected cell. For example, it was reported that in the cells with an AKT-mediated increase in ROS production, FOXM1 becomes a critical survival factor due to its ability to sustain the expression of ROS-detoxifying enzymes [53]. In the current study, we observed that genetic targeting of XBP1 is significantly more deleterious to cells with hyperactive AKT (Figure 4B). XBP1 production is potentially targetable through the chemical inhibition of IRE1α, and numerous compounds with such activity have been described [54,55,56,57,58,59]. However, IRE1α has been reported to carry out other important functions, such as housekeeping control of mitochondrial calcium uptake [60]. Thus, it remains to be seen whether there is a sufficient therapeutic window for safe IRE1α inhibition in a clinical setting. Another concern is the role of XBP1 in immunity. For example, while resting NK cells harbor appreciable amounts of spliced XBP1 transcript, there is a significant increase in the amounts of the accumulated XBP1s protein upon stimulation, and this protein is critical for both the survival and the function of these cells [61]. Interestingly, it was suggested that the additional accumulation of the XBP1s protein in these cells is AKT-dependent, but happens through a post-translational mechanism [61]. It remains to be seen whether this phenomenon is particular to the specialized cell type or has a broader applicability. Finally, it is worth noting that while hyperactive AKT imparts proteotoxic stress and XBP1 reduces proteotoxicity, it remains theoretically possible that other functions of these proteins also contribute to the heightened dependence on XBP1 in conditions of AKT hyperactivation.

Of note, there is considerable interest in the pharmacological targeting of various other components of the proteotoxic stress response [62]. This includes several PERK kinase inhibitors [63,64,65] and the inhibitors of ATF4 function [66,67,68]. At present, these compounds remain investigational tools, and some concerns exist about their potency and off-target effects [62]. 

Another category of potentially relevant compounds is the inhibitors of HSP proteins. Currently, there are dozens of known inhibitors of HSP90, which differ in their potency, mechanisms of action, isoform specificity, and bioavailability [69]. There are some promising observations from early clinical testing of HSP90 inhibition in otherwise highly drug-resistant cancers [70]. However, it may be difficult to separate the anti-cancer effect of such inhibitors that stems from unmitigated proteotoxic stress from more specific effects on cognate HSP90 “client” proteins. The picture is further complicated by the compensatory elevation of HSP70 levels that follows the inhibition of HSP70 [71]. In turn, there are numerous reported HSP70 inhibitors, which also differ in isoform specificity and other chemical and biological traits [72]. While an early trial of an HSP70 inhibitor (MKT-077) failed due to poor bioavailability and high renal toxicity [73], there is considerable effort towards developing HSP70 inhibitors with improved pharmacological properties [72]. Distinct roles of different HSP70 isoforms and their different susceptibility to different inhibitors add to the complexity of the system, which is already complicated by the participation of the HSP70 proteins in both the general response to proteotoxicity and in specific signaling cascades. 

While it remains to be examined whether any of the above-mentioned compounds or combinations thereof are selective against cells with AKT-induced proteotoxic stress, it is encouraging that, at least in some models, the cells with AKT-dependent accumulation of HSP70 are indeed sensitive to HSP70 inhibitors [40]. Additional combinations of these compounds with mild hyperthermia may also be worth exploring.

Importantly, while most of the cells used in our study were not derived from tumors, the sensitizing effect of hyperactive AKT was also noticeable in human melanoma-derived A375 cells (Figure 5). This observation is particularly interesting, as this form of AKT protects these cells from clinically used inhibitors of the MAP kinase cascade [37]. Thus, it appears that the acquisition of resistance to one type of treatment may open up a vulnerability to another. It is yet to be seen how common this phenomenon is. Indeed, it is probable that, during tumor evolution, there is a strong selective pressure towards mitigating AKT-induced stress without diminishing the pro-tumorigenic functions of this enzyme. Therefore, in any individual tumor, it is likely that a combination of additional factors would determine whether the net effect of hyperactive AKT is pro- or anti-apoptotic under the conditions of proteotoxic stress. The identification of such factors is an important subject for future research, as their therapeutic manipulation may help in the selective sensitization of cells that carry some of the most common oncogenic abnormalities.

## 4. Materials and Methods

### 4.1. Cell Culture

All cells were maintained in DMEM medium supplemented with penicillin (100 U/mL), streptomycin (100 µg/mL), and 10% fetal bovine serum. All cultures were kept at 37 °C in the presence of 5% CO_2_ for normal growth conditions. The 293T cells were obtained from the ATCC. MEF HSF1-/- cells were a gift from Dr. Richard Morimoto (Northwestern University). A mouse embryo homozygous for floxed PTEN [31] was provided by the Transgenic Mouse Models Core of the Roswell Park Comprehensive Cancer Center. A culture of mouse embryonic fibroblasts (MEF) was established from this embryo via standard procedures [74] and immortalized using a dominant negative p53 fragment (GSE56), as described previously [75]. Spontaneously immortalized MEF-AG and p53-deficient MEF-P53-/- cells were gifts from Dr. Andrei Gudkov (Roswell Park Comprehensive Cancer Center). 

All cell cultures were routinely verified to be free of mycoplasma, as assessed by MycoAlert Kit (LT07-318; Lonza, Walkersville, MD, USA). 

### 4.2. Plasmids and Virus Production

Lentiviral vectors pLM-CMV-neo and its derivative pLM-CMV-mAKT (expressing a myristoylated form of AKT1) were gifts from Dr. Peter Chumakov (Engelhardt Institute of Molecular Biology). An MLV-based vector pBabePuroMyrAkt [8] was used to express the myristoylated AKT1 in A375 cells, with pBabePuro [76] serving as an empty vector control. The XBP1 shRNA was obtained from Dharmacon (#V2LHS_18218) in the pGIPZ lentiviral backbone. 

pBabeHygroCre vector for the expression of Cre recombinase [77] was used to induce PTEN deletion in PTEN-floxed MEF to generate the MEF PTEN-/- cell line, while pBabeHygro [76] was used as the respective control to generate MEF WT.

The lentiviral expression constructs were packaged in 293T via co-transfection with psPAX2 (from Didier Trono) and pCMV-VSV-G (from Bob Weinberg), which were procured from Addgene (plasmid # 12260 and # 8454). Lentivirus production was performed as described previously [78]. MLV-based constructs were packaged in 293T cells via co-transfection with either pCL-10A1 or pCL-Eco [79] (both from Imgenex, Inc) for the infection of human and mouse cells, respectively. All infections were done by overnight exposure of target cells to viral supernatants, which were filtered through a 0.22 µM PES membrane and supplemented with 5 µg/mL polybrene (Aldrich #107689). Pure cultures of engineered cells were established upon selecting the infected cells for the vector-encoded resistance to the appropriate antibiotic until complete cell death was observed in identically treated uninfected cultures. Each transduction experiment resulted in polyclonal cultures containing >10^3^ independent infectants.

For the measurements of HSF1 transcriptional activity, a construct (pMLucHSP70) expressing Renilla luciferase under the control of human HSP70B promoter HRE-luciferase construct was used essentially as described previously [80]. Reporter assays were conducted 72 h post-transfection. 

### 4.3. Heat Shock

Heat shock was performed via full submersion of tissue culture plates in a thermostated water bath (Precision Water Bath model 286, Thermo Fisher Scientific, Waltham, MA, USA) for the indicated time durations at the indicated temperatures. Plates were made water-tight by wrapping them in Parafilm for the duration of the treatment. 

### 4.4. Comparison of Cell Numbers

Cell numbers were compared using the methylene blue staining and extraction procedure. Cells were fixed with 100% MeOH for 10 min and stained for 15 min with 1% methylene blue in 50% MeOH. The plates were thoroughly rinsed with distilled water and air-dried. Methylene blue dye was extracted using 10% SDS and the absorbance was read at 595 nM. Background values were measured by applying the procedure to unseeded wells. Upon background correction, the signals from experimental plates were compared to those from parallel untreated controls and cells harboring the corresponding control vectors, as indicated. Experiments were carried out in triplicate.

For cell growth assays, the cultures were plated at equal densities and fixed 24, 48, 72, and 96 h after plating. The remaining cells were scored at the indicated time points using the methylene blue staining and extraction procedure. Quadruplicate cultures of each cell variant were analyzed for each time point.

### 4.5. Apoptosis Assays

For the measurement of apoptosis, the Apo-ONE^®^ Homogeneous Caspase-3/7 Assay kit (cat. #G7792) was obtained from Promega Corporation (Madison, WI, USA) and used according to the manufacturer’s recommendations. 

### 4.6. Quantitative RT-PCR

RNA was extracted using the Qiagen RNA Isolation Kit (#74104) according to the manufacturer’s protocols. The reactions were performed and analyzed as described before [81]. All XBP1 qPCR reactions used the same AS primer 5′-GTCCATGGGAAGATGTTCTGG. To detect spliced XBP1, we used the sense primer 5′-CTGAGTCCGAATCAGGTGCAG; for the unspliced XBP1, we used the sense primer 5′-CAGCACTCAGACTATGTGCA; to detect total XBP1, we used the sense primer 5′- TGGCCGGGTCTGCTGAGTCCG. GAPDH expression was used for normalization and detected using Fwd 5′- GTCTCCTCTGACTTCAACAGCG and Rev 5′-ACCACCCTGTTGCTGTAGCCAA primers.

### 4.7. Immunoblotting

Cells were lysed in RIPA buffer (Thermo Fisher Scientific #89900) supplemented with a protease and phosphatase inhibitor cocktail (#5872) from Cell Signaling Technology (Danvers, MA, USA). Cells were taken through a freeze–thaw cycle to ensure complete lysis. The soluble fraction was taken to determine protein concentrations using the Bio-Rad Protein Assay Dye Reagent Concentrate (cat #500-0006) from Bio-Rad Laboratories, Inc. (Hercules, CA, USA). 

For Western blotting, antibodies were diluted according to the manufacturer’s recommendations in BSA and incubated with the membranes overnight at 4 °C with slight agitation. The following primary antibodies were used: AKT (#9272S) and pAKT^s473^ (#4060S) from Cell Signaling; HSP70 (#SC-24), PTEN (#SC-7974), and HPSP90 (#SC-1055) from Santa Cruz; glyceraldehyde 3-phosphate dehydrogenase (GAPDH; #AM4300) from Ambion. 

### 4.8. Data Analysis

Student’s *t*-test was used for pairwise comparisons. Regression analysis of cell growth data and ANOVA analysis with Tukey’s test were done using GraphPad Prism version 9 (GraphPad Software, LLC, San Diego, CA, USA). Image analysis was done using ImageJ [82].

## Supplementary Materials

The following are available online at https://www.mdpi.com/article/10.3390/ijms222111376/s1, Figure S1: Activated AKT sensitizes p53-deficient MEF cells to heat shock.
