# Supplementary figures and images for: Proteotoxic Stress as an Exploitable Vulnerability in Cells with Hyperactive AKT
Source: Int J Mol Sci. 2021 Oct 21;22(21):11376. doi: 10.3390/ijms222111376 (PMC8583472; doi:10.3390/ijms222111376)

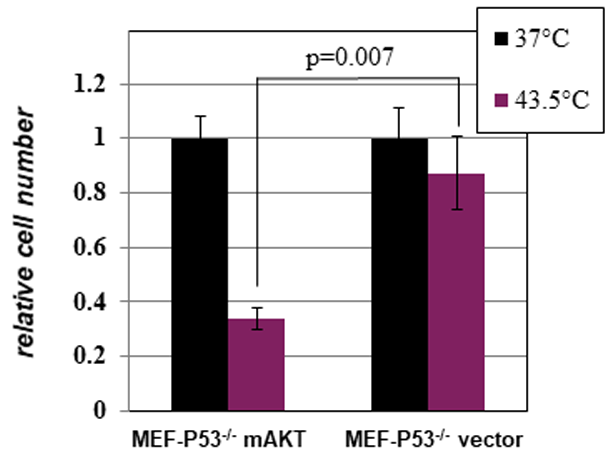

Supplement: Supplementary file 1 [file ijms-22-11376-s001.zip › Suppl FigureS1.tif]
